# Small-scale mechanical behavior of a eutectic high entropy alloy

**DOI:** 10.1038/s41598-020-59513-2

**Published:** 2020-02-14

**Authors:** Saideep Muskeri, Vahid Hasannaeimi, Riyadh Salloom, Maryam Sadeghilaridjani, Sundeep Mukherjee

**Affiliations:** Department of Materials Science and Engineering, University of North Texas, Denton, TX 76203 USA

**Keywords:** Structural materials, Techniques and instrumentation

## Abstract

Eutectic high entropy alloys, with lamellar arrangement of solid solution phases, represent a new paradigm for simultaneously achieving high strength and ductility, thereby circumventing this well-known trade-off in conventional alloys. However, dynamic strengthening mechanisms and phase-boundary interactions during external loading remain unclear for these eutectic systems. In this study, small-scale mechanical behavior was evaluated for AlCoCrFeNi_2.1_ eutectic high entropy alloy, consisting of a lamellar arrangement of L1_2_ and B2 solid-solution phases. The ultimate tensile strength was 1165 MPa with ductility of ~18% and ultimate compressive strength was 1863 MPa with a total compressive fracture strain of ~34%. Dual mode fracture was observed with ductile failure for L1_2_ phase and brittle mode for B2 phase. Phase-specific mechanical tests using nano-indentation and micro-pillar compression showed higher hardness and strength and larger strain rate sensitivity for B2 compared with L1_2_. Micro-pillars on B2 phase deformed by plastic barreling while L1_2_ micro-pillars showed high density of slip steps due to activation of more slip systems and homogenous plastic flow. Mixed micro-pillars containing both the phases exhibited dual yielding behavior while the interface between L1_2_ and B2 was well preserved without any sign of separation or cracking. Phase-specific friction analysis revealed higher coefficient of friction for B2 compared to L1_2_. These results will pave the way for fundamental understanding of phase-specific contribution to bulk mechanical response of concentrated alloys and help in designing structural materials with high fracture toughness.

## Introduction

Multi-principal element alloys (MPEAs), also known as high entropy alloys (HEAs), offer a new paradigm in structural alloy design and development owing to their appealing physical and mechanical properties such as high fracture toughness, good wear and fatigue resistance, high strength and good elevated temperature properties^1–3^. Several HEA systems have been reported with a single-phase face center cubic (FCC) or body center cubic (BCC) structure^4–6^. To concurrently achieve good ductility of FCC HEAs along with high strength of BCC HEAs, eutectic high entropy alloys (EHEAs) have recently been developed to overcome the limitations of single-phase HEAs^7^. EHEAs typically consist of a mixture of two solid-solution phases in the form of lamellae or rod-like dispersions in a matrix. To date, several EHEAs have been reported with excellent combination of high strength and ductility including CoFeNi_1.4_VMo^8^, Co_2.0_Mo_0.8_Ni_2.0_VW_0.8_^9^, CoFeNi_2.0_V_0.5_Nb_0.75_^10^, CoCrFeNiTa_0.4_^11^, CoCrFeNiNb_0.65_^12^, Fe_20_Co_20_Ni_41_Al_19_^13^, CoCrFeNiTa_x_^14^, AlCoCrFeNb_x_Ni^15^, AlCoCrFeNiZr_x_^16^, and AlCoCrFeNi_x_^17^. Among all the aforementioned EHEAs, AlCoCrFeNi_2.1_ alloy, with lamellar arrangement of BCC (B2) and FCC (L1_2_) solid-solution phases has shown the most promising properties that can be tuned over a large range with thermo-mechanical processing. The bulk tensile and compressive behavior of as-cast and thermo-mechanically processed AlCoCrFeNi_2.1_ has been reported along with detailed microstructural analysis^18–21^. However, dynamic strengthening mechanisms and phase interactions during external loading is not well understood for these eutectic alloys. The large fraction of phase boundary significantly impacts plastic flow in these systems. Considering the effect of thermo-mechanical processing on tailoring the microstructure and mechanical properties of AlCoCrFeNi_2.1_ EHEA^18–21^, understanding of phase-specific mechanical response is critical for fundamental insights as well as wider application of these alloys. Here, phase-specific mechanical behavior of AlCoCrFeNi_2.1_ eutectic high entropy alloy was evaluated for better understanding of phase boundary interactions during loading. Measurement of hardness, modulus, strain rate sensitivity, yielding and plastic deformation of micro-pillars, and friction analysis was done at the microstructural length-scale and compared with bulk mechanical response. This will pave the way for designing stronger and tougher complex phase HEAs.

## Experimental

The AlCoCrFeNi_2.1_ eutectic high entropy alloy was prepared by melting weighted proportion of pure elements (>99.9% purity) in a vacuum arc-melter and homogenized through multiple flips and re-melting cycles. The samples were polished to a surface roughness of 0.02 µm using colloidal silica for electron microscopy characterization. The microstructures were recorded using FEI Quanta scanning electron microscope (SEM). Energy dispersive X-ray spectroscopy (EDS) and electron backscatter diffraction (EBSD) analysis were carried out using FEI Nova Nano SEM230. Transmission electron microscopy (TEM) foils were prepared with FEI Nova NanoLab 200 dual-beam focused ion beam (FIB) in multiple steps starting with 30 kV Ga^+^ ions down to 5 kV ions for reducing surface damage induced by the high energy ions. A Philips EM-420 operating at 120 kV was used to perform the TEM imaging.

Mini-tensile specimens were prepared using electrical discharge machining (EDM) with a gauge length of ~5 mm and width of ~1 mm. Both sides of the specimen were polished using SiC paper to achieve a final thickness of ~0.9 mm. Bulk compression samples were prepared with a diameter of 3 mm and height of 5 mm and both the top and bottom surfaces were polished to achieve flat surfaces. An Instron 4482 (Instron, Norwood, MA, USA) was used to perform bulk compression tests and a computer-controlled tensile testing frame with 500 pound load cell was used to perform mini-tensile tests. Mini-tensile and bulk compression tests were performed at a strain rate of 10^−3^ s^−1^. At least three samples were tested in tension and compression to determine the average and standard deviation.

Nano-indentation tests were done using TI-Premier Triboindenter (Bruker, Minneapolis, MN, USA) using a diamond Berkovich tip at room temperature. A maximum load of 10000 µN was used for phase-specific hardness measurements. During each indent, the load was linearly increased to 10000 µN over a period of 5 s, held constant for 2 s and unloaded in 5 s. Initially, an identification mark was made on the sample followed by a series of 10 × 10 square grids. Subsequently, phase-specific indents were identified in SEM and their corresponding hardness and modulus values were determined. The average values of hardness and modulus were obtained from at least 15 indents on each phase. High speed nano-indentation mapping tests were performed at a maximum load of 500 µN. Partial-unload nano-indentation mode was used to study the phase specific indentation size effect (ISE). Indents were made at various loads ranging from 350 µN to 10000 µN and corresponding hardness was recorded at each load. Phase specific strain rate sensitivity (SRS) was also investigated by nano-indentation at a load of 3000 µN at three different strain rates of 4 × 10^−2^ s^−1^, 1.2 × 10^−1^ s^−1^ and 4 × 10^−1^ s^−1^. For ISE and SRS measurement, arrays of 10 × 5 were made and the indents on each phase were identified by SEM for further analysis. For comparison, strain rate sensitivity of the bulk sample was studied at a high load of 5 N to cover several grains with different orientations to be representative of the bulk behavior. For all nano-indentation tests, a separation of 10 times the indent size was maintained to avoid the overlapping of plastic deformation from the adjacent indents.

Grain orientations were determined by Orientation Imaging Microscopy (OIM) using FEI Nova Nano SEM230. Micro-pillars were prepared on selected orientations with FEI Nova NanoLab 200 focused ion beam (FIB) in several steps using a Ga beam current ranging from 5 nA to 10 pA at 30 kV. The final diameter of all micro-pillars was 2.0 ± 0.3 µm with aspect ratio (height to diameter) of ~2. At least three micro-pillars were made on different regions to ensure consistency in results. The micro-pillars were uniaxially compressed using PI88 SEM pico-indenter (Bruker, Minneapolis, MN, USA) with a load cell of 500 mN in displacement-control mode. A flat diamond punch with a diameter of 5 µm was used for compression. All the micro-pillars were compressed at a constant strain rate of ~2.5 × 10^−4^ s^−1^. During the compression tests, videos were recorded with a frame rate of 0.1 frames/s. Pillar dimensions were measured based on SEM images corrected for the taper angle. Using the initial diameter and height of the pillar, load-displacement data was converted to engineering stress-strain. Phase-specific scratch behavior was studied using the PI88 SEM pico-indenter with a load of 500 mN. A diamond cube-corner tip was used to perform the scratch across the two phases with a ramping load up to 3000 µN. At least 6 scratches were performed in different regions for statistical analysis.

## Results and Discussion

### Microstructural characterization

**Figure 1:**
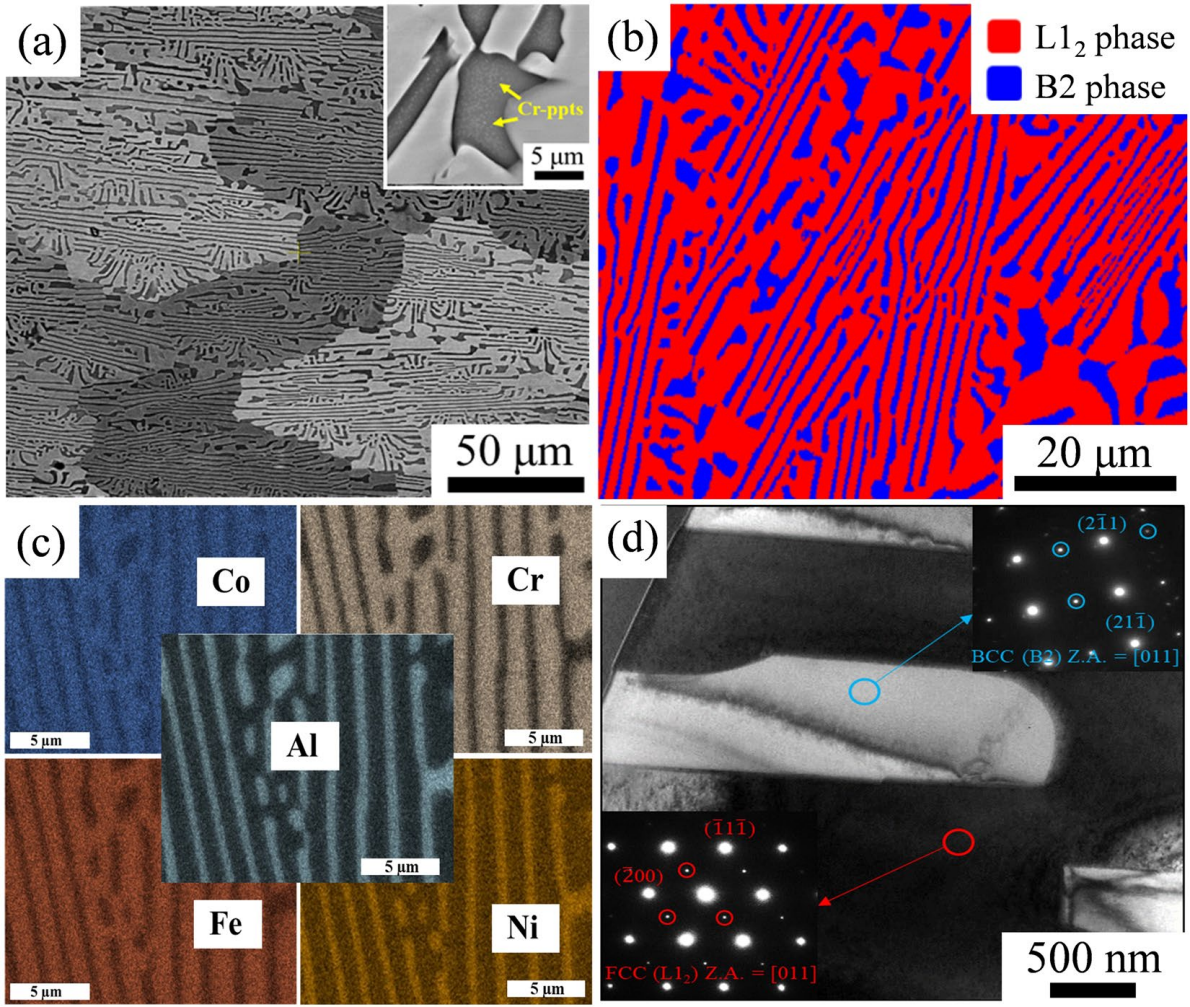
(**a**) Back scattered electron micrograph of as-cast AlCoCrFeNi_2.1_ alloy; (**b**) EBSD phase map showing the eutectic lamellar structure with blue and red colors for B2 and L1_2_ phases, respectively; (**c**) EDS map showing B2 phase was rich in (Ni and Al) and L1_2_ phase rich in (Co, Cr and Fe); (**d**) TEM bright field image showing the phase contrast for the lamellar structure with the selected area diffraction patterns as insets. The zone axis [Z.A] for measurements are indicated in the insets.

The as-cast microstructure of AlCoCrFeNi_2.1_ EHEA is shown in Fig. 1(a). The microstructure consists of lamellar arrangement of L1_2_ (light contrast) and B2 (dark contrast) solid-solution phases with an average grain size of ~100 µm. The B2 phase consisted of nano-sized particles (white spots) which are shown in the high magnification image (inset in Fig. 1(a)). Previous studies have shown these nano-sized precipitates to be Cr-rich^22,23^. EBSD phase map in Fig. 1(b) shows the lamellar structure with L1_2_ (red color) and B2 (blue color) phases. The B2 lamellae with thickness of ~2 µm are distributed in the L1_2_ matrix along with some coarse B2. The volume fraction of the L1_2_ phase was determined to be approximately 71% and B2 phase was 29%. The EDS map of the as-cast microstructure is shown in Fig. 1(c). The B2 phase was found to be rich in Ni and Al while the L1_2_ phase was rich in Co, Cr, and Fe. Bright field TEM micrograph in Fig. 1(d) shows the lamellar microstructure with distinct contrast between B2 (bright) and L1_2_ (dark) phases. TEM-selected area diffraction patterns (insets in Fig. 1(d)) show the presence of superlattice spots which confirm the ordered L1_2_ and B2 structure, consistent with reported literatures^21,23,24^.

### Bulk-tensile and compression

**Figure 2:**
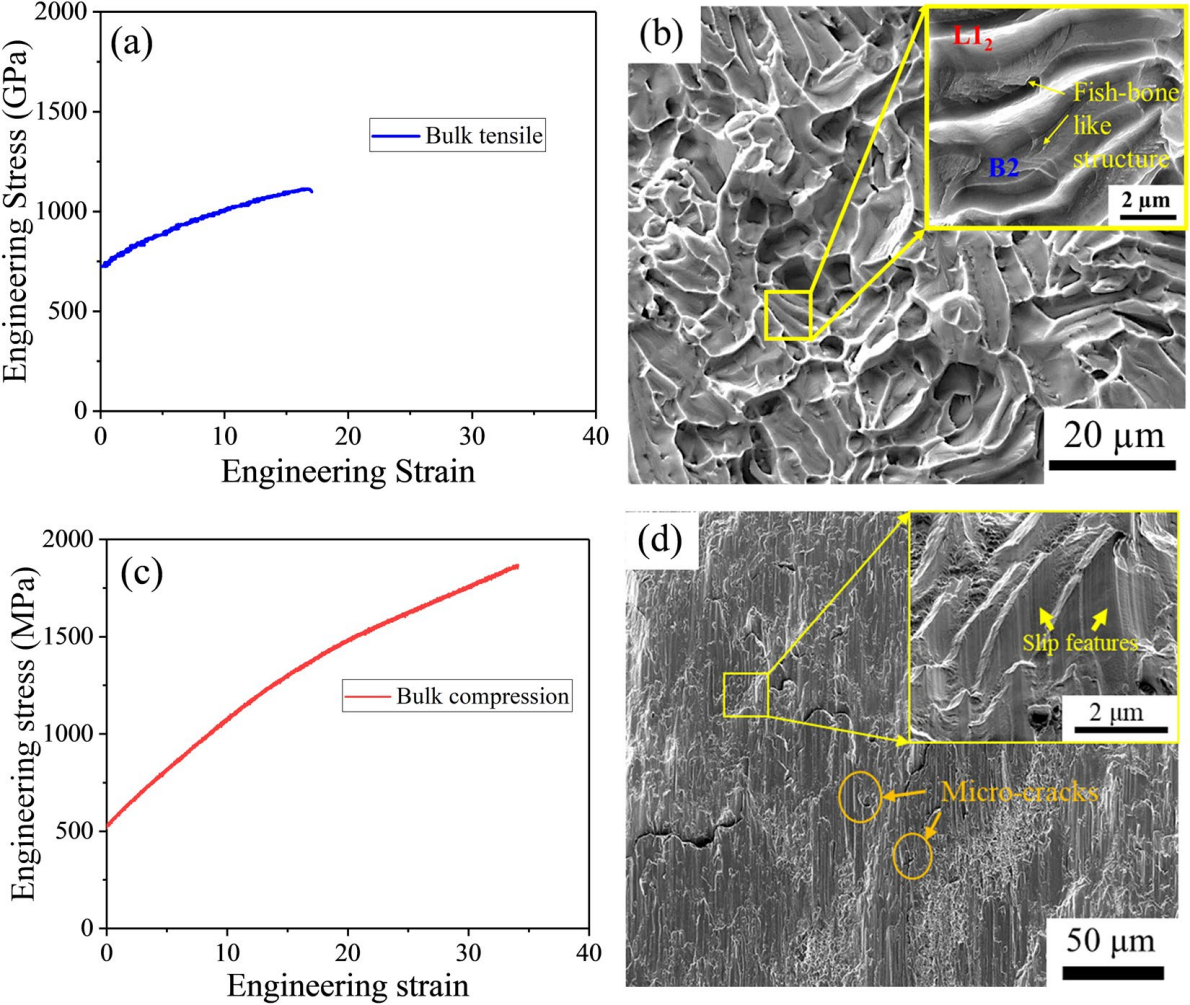
(**a**) Engineering tensile stress-strain curve of the as-cast AlCoCrFeNi_2.1_ alloy; (**b**) fracture morphology after bulk tensile test showing dual mode fracture with ductile failure for L1_2_ phase and brittle failure for B2 phase; (**c**) Bulk compression stress-strain curve of the as-cast AlCoCrFeNi_2.1_ alloy; (**d**) fracture morphology after bulk compression test showing quasi-cleavage type of fracture.

Tensile engineering stress-strain plot of the as-cast alloy is shown in Fig. 2(a). The EHEA showed a yield strength (σ_0.2_) of 750 ± 24 MPa, ultimate tensile strength (σ_UTS_) of 1165 ± 20 MPa and elongation of ~18%. The AlCoCrFeNi_2.1_ EHEA showed a better combination of strength and ductility in tension when compared to several other reported EHEAs^13,17^. The fracture morphology of the sample after tensile test is shown in Fig. 2(b). The fracture surface showed two different fracture features. The B2 phase fractured in a brittle manner with the cracks initiating at one end and propagating to the other by leaving fish-bone shaped markings with radial stripes. In contrast, the L1_2_ phase fractured in a ductile manner by necking into sharp lines without forming any dimples^23^. The compressive engineering stress-strain curve of AlCoCrFeNi_2.1_ EHEA is shown in Fig. 2(c). The alloy showed a compressive yield strength (σ_0.2_) of 517 ± 7 MPa and compressive ultimate strength (σ_UTS_) of 1863 ± 12 MPa, with a total compressive fracture strain of ~34%. These compressive properties are superior to the other eutectic compositions as well^14,25^. The compressive fracture surface is shown in Fig. 2(d) with cleavage steps and slip features that are clear in the higher magnification image (inset). These features suggest quasi-cleavage type of fracture for AlCoCrFeNi_2.1_ EHEA in the compression mode, with micro-cracks originating at the coarse B2 phase and deflecting from the interface to grow into large cracks.

The AlCoCrFeNi_2.1_ alloy showed significant tension-compression asymmetry similar to that reported for Mg alloys^26^, NiTi superalloys^27^, and titanium alloys^28^. In a eutectic alloy, the morphology and orientation of eutectic lamellae with respect to the loading direction significantly influence the mechanical properties. Due to the different cooling conditions in the eutectic ingot, the orientation of the eutectic colonies may have resulted in anisotropic behavior depending on the location from which the test samples were taken^29^. Another factor contributing to tension-compression asymmetry may be the difference in elastic properties between the matrix (L1_2_ phase) and secondary phases (B2 phase), which may have caused internal stresses (Bauschinger effect)^30^. Finally, the volume fraction of ductile L1_2_ matrix and brittle B2 phase may have contributed to the asymmetric behavior as well.

### Small-scale mechanical behavior by nano-indentation

#### Hardness and modulus

**Figure 3:**
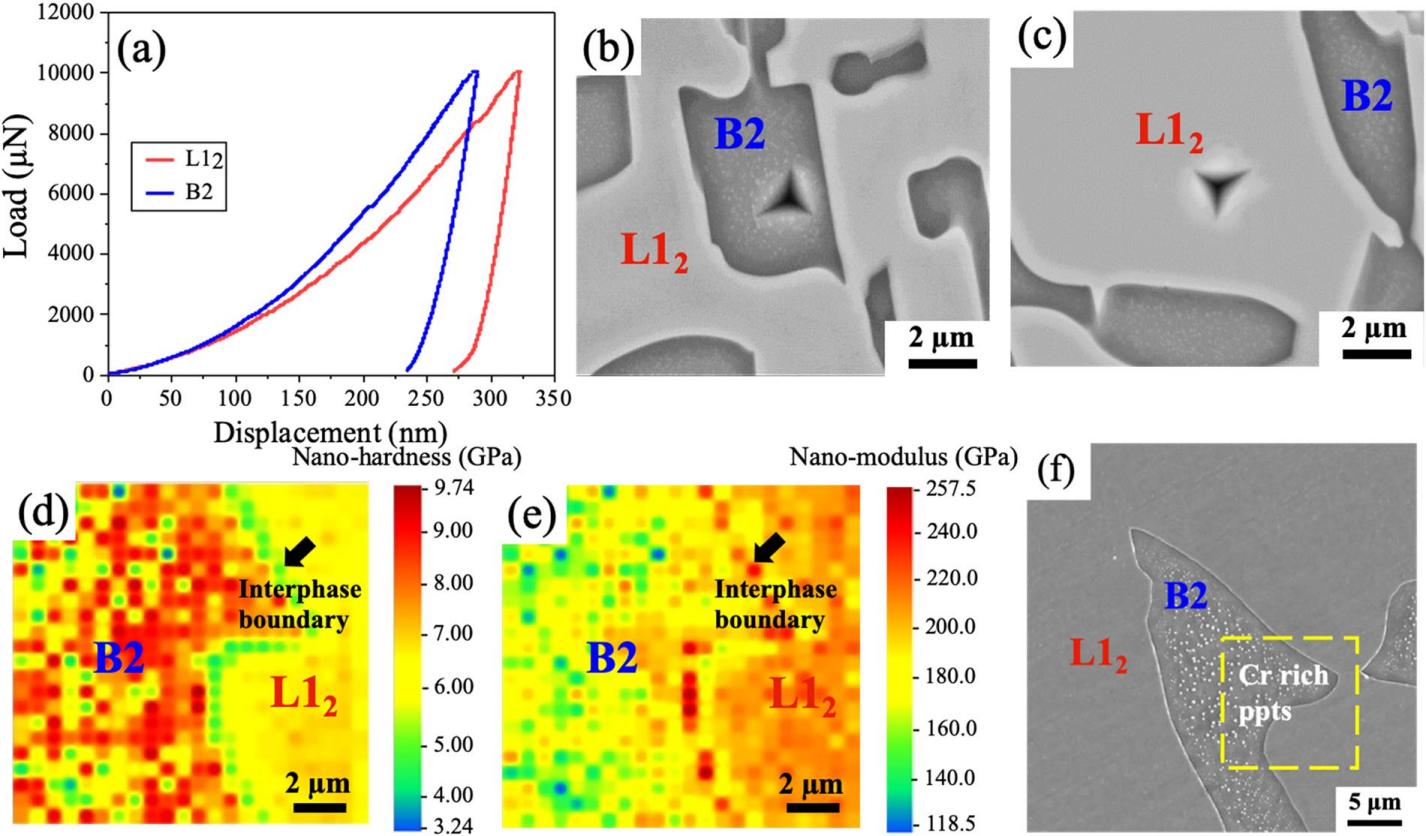
(**a**) Load-displacement curves for L1_2_ and B2 phases; (**b**) SEM image of indent on B2 phase; (**c**) SEM image of indent on L1_2_ phase; (**d**) Nano-indentation hardness map showing B2 phase on left and L1_2_ phase on right; (**e**) Nano-indentation modulus map showing B2 phase on left and L1_2_ phase on right; (**f**) SEM image showing the location of the nano-indentation mapping marked with yellow box.

Small-scale mechanical tests were performed to evaluate the phase specific contribution to bulk mechanical behavior. Nano-indentation tests were carried out at the load of 10000 µN to compare the hardness and modulus of the individual B2 and L1_2_ phases. Representative load-displacement curves of B2 and L1_2_ phases are shown in Fig. 3(a). The B2 phase showed lower peak displacement (~270 nm) when compared to L1_2_ phase (~325 nm). The location of indents on B2 and L1_2_ phase are shown in Fig. 3(b),(c), respectively. A minimum of 15 indentations on each phase were performed to obtain average and standard deviation of hardness and modulus. The hardness and modulus values of individual phases of as-cast alloy are summarized in Table 1.

**Table 1:** Average phase-specific hardness and modulus values from nano-indentation.

Phase	Average hardness (GPa)	Average modulus (GPa)
B2	4.91 ± 0.71	180.07 ± 10.34
L1_2_	4.03 ± 0.40	216.05 ± 23.89

High-speed nano-indentation mapping was used at a low load (500 µN) to evaluate the hardness and modulus distribution in each phase as well as the phase boundary. A separation of 10 times the indent size was maintained to avoid the overlapping of plastic deformation from adjacent indents. It is evident from Fig. 3(d) that B2 phase has higher hardness compared to L1_2_ phase. The corresponding modulus map is shown in Fig. 3(e). The location in the SEM image for the nano-indentation maps is shown in Fig. 3(f) marked with a yellow box. The B2 phase showed an average hardness of 7.5 ± 2.2 GPa at the load of 500 µN whereas the L1_2_ phase showed an average hardness of 6.0 ± 0.5 GPa. The inter-phase boundary (marked with black arrow) showed an average hardness of 5.0 ± 0.2 GPa, lower than that of the individual phases in the as-cast alloy. The hardness and modulus values showed a much more homogenous distribution for the L1_2_ phase compared to B2. This may be attributed to the presence of the Cr-rich precipitates in B2 phase. These Cr-rich precipitates appear to be slightly softer and less stiff compared to the B2 matrix as seen from Fig. 3(d),(e).

#### Strain gradient plasticity and strain rate sensitivity

From Table 1 (hardness at 10000 μN) and Fig. 3(d) (hardness at 500 μN), it can be seen that the hardness values of both B2 and L1_2_ phases were lower at higher loads, which is attributed to strain gradient plasticity^31–33^. This depth dependence of hardness arises from geometrically necessary dislocations (GNDs), which are generated to accommodate the shape of the indenter during nano-indentation. GNDs restrict the motion of statistically stored dislocations (SSDs) leading to strain hardening effect^34^, with the density of GNDs being inversely proportional to the indentation depth^31,35^.

**Figure 4:**
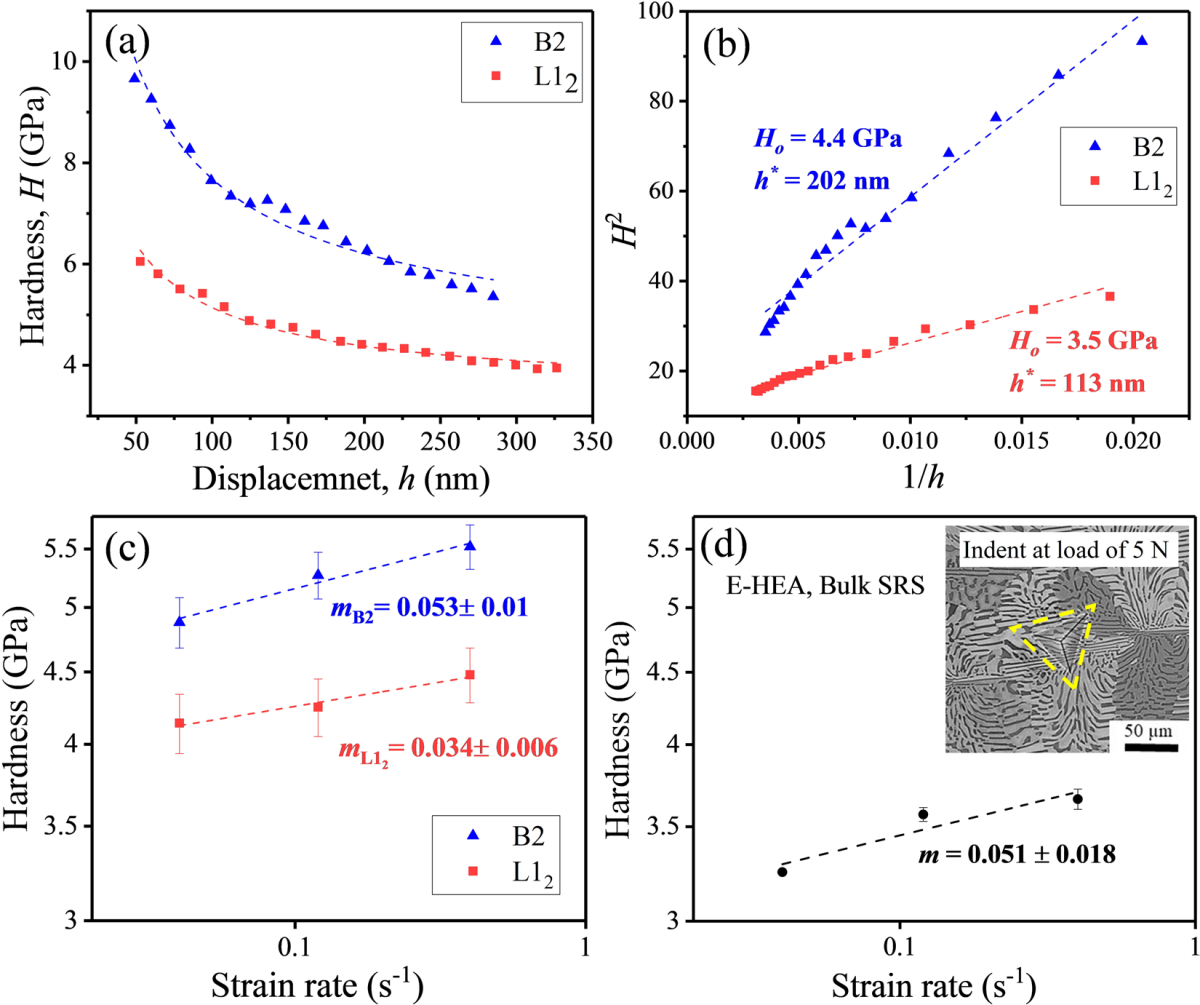
(**a**) Hardness dependence of depth for the two phases in as-cast AlCoCrFeNi_2.1_ alloy, B2 and L1_2_; (**b**) H^2^ versus 1/h shows characteristic length scale (h*) for B2 and L1_2_ phases; (**c**) hardness as a function of strain rate at a load of 3000 μN in double logarithmic scale for both the phases showing higher strain rate sensitivity for B2 phase; (**d**) hardness dependence on strain rate at a load of 5 N to be representative of bulk sample.

Indentation size effect (ISE) for B2 and L1_2_ phases was analyzed using loads in the range of 350 μN to 10000 μN. Hardness as a function of depth for each phase is shown in Fig. 4(a). It was found that the hardness decreased with increasing depth for both B2 and L1_2_ phases. The hardness, *H*, was correlated with depth, *h*, according to Nix/Gao model as^31^:

1 $$\frac{H}{{H}_{0}}=\sqrt{1+\frac{{h}^{\ast }}{h}}$$

where, *H*_*o*_ is the hardness independent of depth and h* is the characteristic length of ISE. Based on Eq. (1), the characteristic length (h*) was calculated from the slope of *H*^2^ versus 1/*h* as shown in Fig. 4(b) and the value of *H*_*o*_ was obtained from the intercept. The *H*_*o*_ and h* values for B2 and L1_2_ phases were calculated and shown in Fig. 4(b). The h* was ~75% larger for B2 phase compared to L1_2_, which may be attributed to the smaller storage volume (*f*) of geometrically necessary dislocations in the B2 phase. The storage volume of GNDs is an area not in contact with the indenter, where the strain level is high enough for plastic deformation^33,35^. The smaller storage volume of GNDs results in a larger hardness difference over a shallower depth and leads to larger h*. The storage volume was calculated from modified Nix/Gao model^32,33^ and was 1.2 for B2 phase and 1.77 for L1_2_. This indicates that the movement of GNDs is more restricted in the B2 phase leading to smaller storage volume and consequently larger h*.

Phase-specific thermally activated deformation in terms of strain rate sensitivity (SRS) of B2 and L1_2_ phases was also investigated. Peak load of 3000 μN was used at the strain rates of 4 × 10^−2^ s^−1^, 1.2 × 10^−1^ s^−1^ and 4 × 10^−1^ s^−1^. An array of 10 × 5 indentations were made and the indents on each phase were identified by SEM similar to Fig. 3(b),(c). The phase-specific hardness values corresponding to each strain rate are plotted in Fig. 4(c) in double logarithmic scale. Strain rate sensitivity *(m)* was calculated from the slope of the linear fitting of the curves and is shown alongside the plots^36^. In ordered BCC phase (B2), deformation is dominated by movement of screw dislocations. However, in ordered FCC phase (L1_2_), dislocation glide (edge or screw or mixed) over obstacles is the dominant mechanism. The B2 phase showed a higher SRS than L1_2_ phase, which is consistent with literature^37–39^. To evaluate the bulk response, overall SRS of the EHEA was determined at a load of 5 N at strain rates of 4 × 10^−2^ s^−1^, 1.2 × 10^−1^ s^−1^ and 4 × 10^−1^ s^−1^. The indent covered a large area and included both the phases covering several grains (Fig. 4(d) inset) to be representative of bulk SRS. The hardness at 5 N as a function of strain rate is shown in Fig. 4(d), with the calculated SRS value very close to that of the B2 phase. This suggests that the stronger B2 phase controls the deformation mechanism in AlCoCrFeNi_2.1_ EHEA. The results are similar to a bulk metallic glass composite, where plastic flow was found to be controlled by the harder matrix rather than softer phase^40^.

### Micro-pillar compression

**Figure 5:**
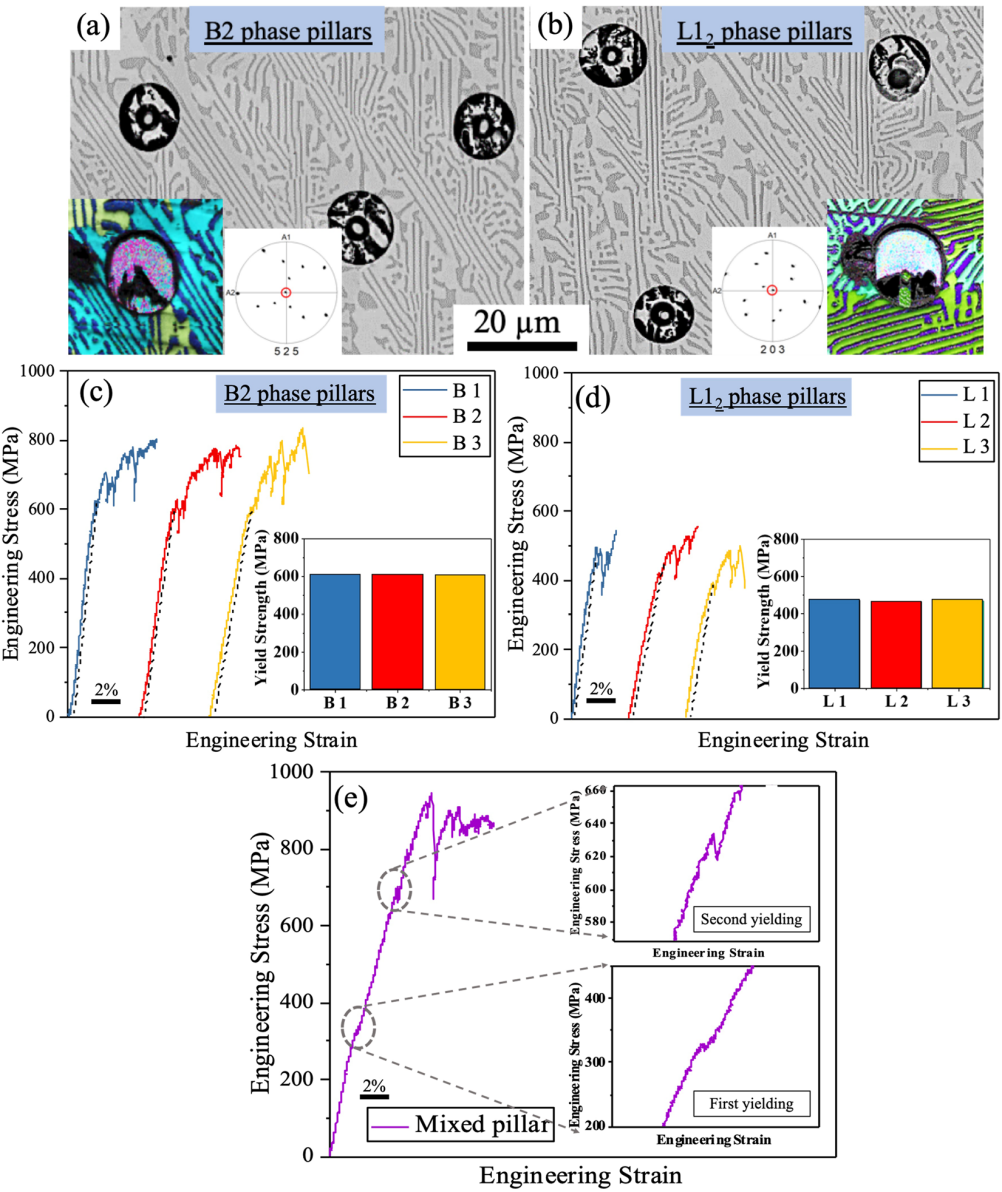
(**a**) SEM image of [525] oriented B2 micro- pillars (inset figure shows IPF + IQ map and a pole figure with a point in center (circled in red) confirms [525] orientation of the B2 pillar); (**b**) SEM image of [203] oriented L1_2_ micro-pillars (inset figure shows IPF + IQ map and a pole figure with a point in center (circled in red) confirms [203] orientation of the L1_2_ pillar); (**c**) compressive engineering stress-strain curves (dotted lines in black indicate 0.2% offset) of the [525] oriented B2 micro-pillars with the inset showing yield strength values for three representative micro-pillars; (**d**) compression engineering stress-strain curves (dotted lines in black indicate 0.2% offset) of the [203] oriented L1_2_ micro-pillars with the inset showing yield strength values for three representative pillars; (**e**) compressive engineering stress-strain curves of a micro-pillar containing both B2 and L1_2_ phases showing double yielding phenomenon.

Phase-specific plastic deformation behavior of the as-cast AlCoCrFeNi_2.1_ was studied by micro-pillar compression under uniaxial load concurrent with *in situ* observation of deformation and failure. This helped in estimating the strength contribution of each phase towards the overall bulk mechanical behavior. The micro-pillars chosen were in the orientation of [525] and [203] for the B2 and L1_2_ phases, respectively. SEM images of micro-pillars on the B2 and L1_2_ phases are shown in Fig. 5(a),(b), respectively. The insets show the pole figure + image quality (IPF + IQ) images of the micro-pillars for both the phases. The points in the pole figures indicate the set of planes for the respective orientations. The point lying in the center of the pole figures (circled in red) confirms the [525] and [203] orientations for B2 and L1_2_ phases, respectively. The compressive engineering stress-strain plots for micro-pillars on B2 and L1_2_ phases are shown in Fig. 5(c),(d), respectively. The yield strength values from the compression of micro-pillars on B2 phase and L1_2_ phase are shown as insets in Fig. 5(c),(d). The B2 phase pillars showed an average yield strength of ~610 MPa while the L1_2_ phase pillars showed an average yield strength of ~460 MPa. The pillars fabricated on B2 and L1_2_ phases had different orientation and yield strength changes with grain orientation. Therefore, the critical resolved shear stress (CRSS) was calculated for each phase using Schmid factor^41–43^ to account for the crystal orientation. The slip system with maximum Schmid factor for B2 phase was (121)[1$\bar{1}$ 1] and for L1_2_ phase were two, namely (111)[0$\bar{1}$ 1] and (1$\bar{1}$ 1)[011]. Converting the yield strength to the critical resolved shear stress for slip system of each phase, the maximum CRSS was 299 MPa for the B2 phase and 221 MPa for L1_2_ phase. The deformation behavior of mixed phase micro-pillars containing both B2 and L1_2_ phases is shown in Fig. 5(e). The stress-strain curve may be divided into three different regimes. Firstly, the L1_2_ phase with ordered FCC crystal structure yielded around 350 MPa. After this point, L1_2_ was plastically deformed while B2 was still in elastic region. This was supported by the slight change in the slope of the stress-strain curve after the first yield point (Fig. 5(e)). Once the load reached close to the yield strength of B2, a second pronounced yield point was observed (~620 to 640 MPa). Following this, there was plastic deformation of both B2 and L1_2_ as the load increased. This double yielding behavior has been reported for several composite materials including bulk metallic glass composites^44^ and polymer micro-frames^45^. However, the multiple yield points were not detected in bulk tensile or compression tests likely due to the small difference between the modulus of B2 and L1_2_ phases (Table 1) which could not be captured by the lower resolution of the bulk experiments.

**Figure 6:**
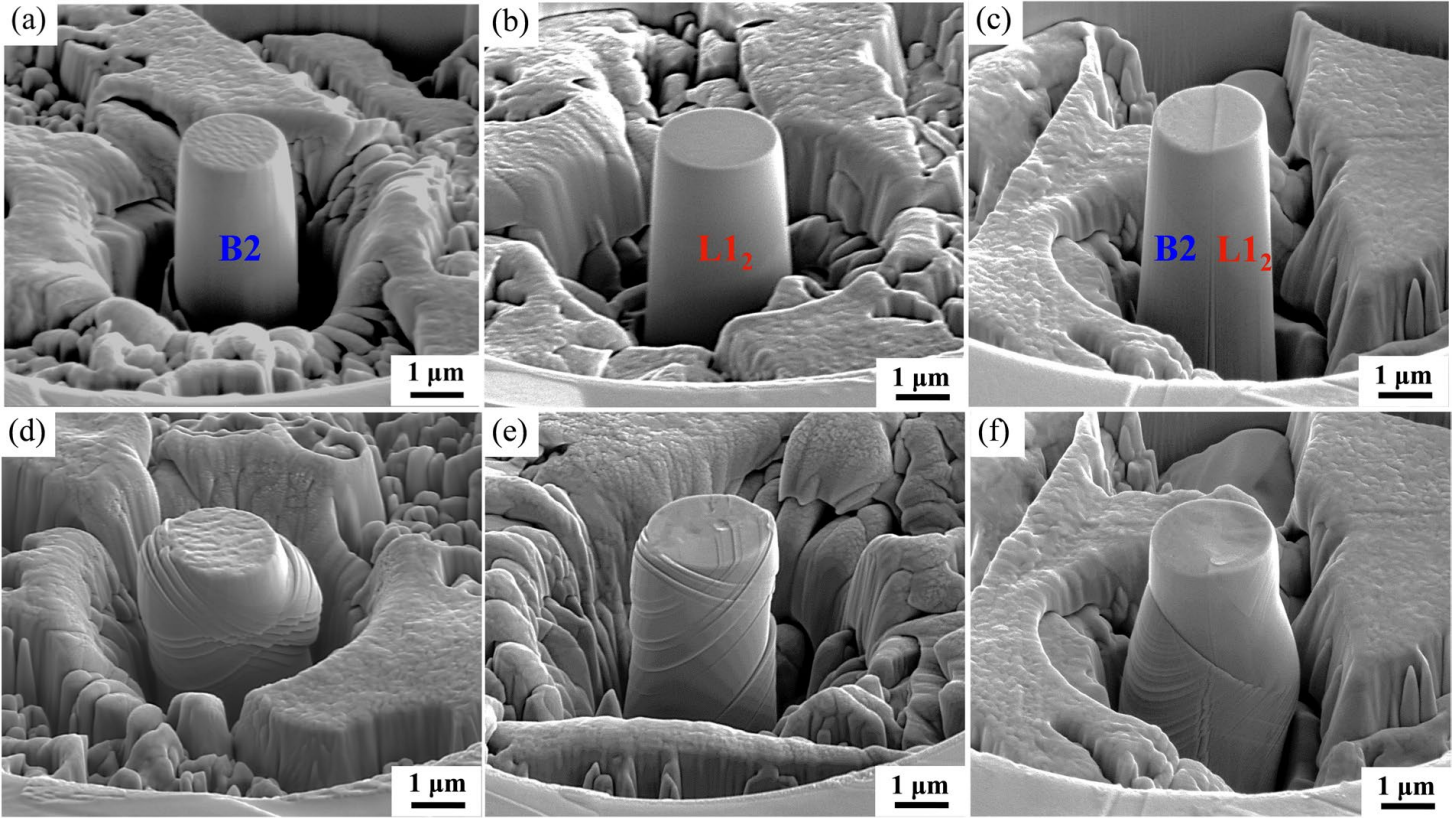
(**a**) SEM image of a [525] oriented B2 micro-pillar before compression showing a rough surface due to the presence of Cr-rich nano-precipitates; (**b**) SEM image of [203] oriented L1_2_ micro-pillar before compression; (**c**) Mixed micro-pillar containing B2 and L1_2_ phases before compression; (**d**) SEM image of [525] oriented B2 micro-pillar after compression showing plastic barreling effect due to limited slip systems and high hardness; (**e**) SEM image of [203] oriented L1_2_ micro-pillar after compression showing high density of slip steps due to the activation of more slip systems and homogenous plastic flow; (**f**) SEM image of mixed micro-pillar after compression.

Figure 6 shows the SEM images of micro-pillars fabricated on the B2 phase (Fig. 6(a)) and L1_2_ phase (Fig. 6(b)) in [525] and [203] crystallographic orientations, respectively. A mixed micro-pillar containing both phases is shown in Fig. 6(c). The top surface of the B2 pillars had a rough appearance due to the presence of Cr-rich nano-precipitates and the B2 deformed as a hard phase reinforced by nano-precipitates^23^ as shown in Fig. 6(d). Due to limited slip systems and higher hardness, majority of B2 micropillars deformed through plastic barreling (Fig. 6(d)). The barreling effect was more apparent in the upper part of the pillar due to friction between the pillar top surface and the indenter^46^. The amount of barreling increased with the applied load followed by formation of slip steps once the barreling load exceeded. In contrast, the micro-pillars in L1_2_ phase after deformation showed a high density of slip steps due to the activation of more slip systems and homogenous plastic flow as shown in Fig. 6(e). The deformation mechanism in conventional FCC alloys is mainly influenced by dislocation slip and twinning^5^. The phase boundary in a dual phase alloy such as EHEA plays an important role in crack nucleation and failure mode. During compression of mixed pillars (Fig. 6(f)), initial slip steps formed near the top of L1_2_ while B2 was still elastically deforming. Plastic deformation of B2 initiated at higher loads while there was continued work hardening of L1_2_^23^. Enhanced plasticity in this EHEA during bulk tensile testing has been explained by complex back-stresses from L1_2_^23^. A similar phenomenon was observed for the mixed micro-pillars. Once B2 plastic deformation initiated, it proceeded with the rapid formation of slip bands and majority of the plastic flow was concentrated on the B2 side since L1_2_ was sufficiently work hardened. At higher loads, both L1_2_ and B2 deformed simultaneously with the initiation and propagation of slip steps but the phase boundary was preserved without any sign of separation or cracking even after high compressive strains. This demonstrates that the semi-coherent phase boundary had a major contribution towards the high strength and ductility of this EHEA.

**Figure 7:**
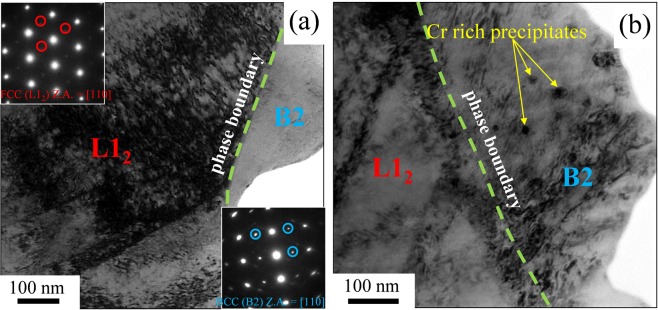
(**a**) Bright-field TEM image of a deformed mixed micro-pillar showing the interface between L1_2_ and B2 with high dislocation density in L1_2_. The insets show the SAD patterns of the two phases along [110] zone axis; (**b**) higher magnification bright-field TEM image of L1_2_/B2 boundary region showing the Cr-rich precipitates within the B2 phase, the phase boundary is indicated by a green dotted line in (**a**,**b**).

TEM analysis was performed on a deformed mixed micro-pillar containing the B2 and L1_2_ phases in order to understand the effect of deformation on the interface after the compression test. Figure 7 shows bright-field TEM images of a mixed micropillar. The B2 and L1_2_ regions are indicated within the bright-field image in Fig. 7(a). The superlattice diffraction spots within the TEM selected area diffraction (SAD) patterns (shown as insets) confirmed that both phases were still ordered after the compression and no disordering and phase transformation occurred after deformation. Significantly higher dislocation density was observed for the L1_2_ phase compared to B2 near the interface supporting the greater extent of plastic deformation for the L1_2._ Zoomed in view near the B2 phase (Fig. 7(b)) showed high density of nano-sized precipitates that were previously identified as Cr-rich with disordered BCC structure^19,23^. The average size of the precipitates was about 20 nm. The presence of these precipitates further enhanced the strength of B2 phase by acting as barriers for dislocation movement. Toughening in HEAs is influenced by the formation of stacking-fault parallelepipeds, arresting of undissociated dislocations at planar slip bands, and easy motion of Shockley partial dislocations^5^. The micro-pillar compression tests revealed that the interphase boundary was preserved during deformation and led to exceptional strain hardening and strength-ductility combination.

### Friction analysis

**Figure 8:**
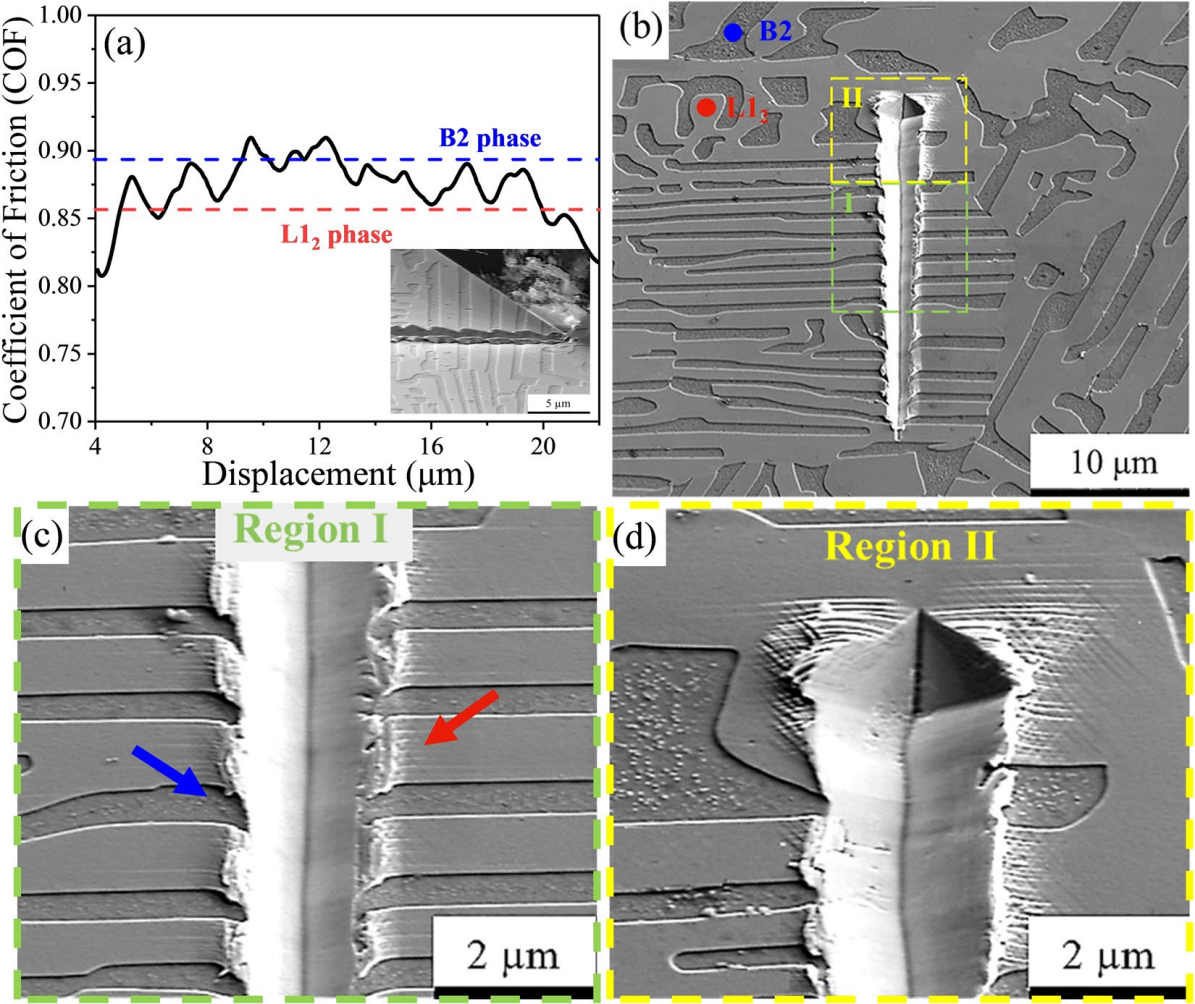
(**a**) Coefficient of friction (COF) versus displacement plot with the inset showing the EHEA and cube corner diamond indenter after the test; (**b**) low magnification SEM image showing scratch along eutectic lamellae; the L1_2_ and B2 phases are marked with red and blue dots, respectively; (**c**) high magnification SEM image of region I showing slip lines extending perpendicular to the scratch direction and more material removal for L1_2_ phase (indicated with red arrow) compared to B2 (indicated with blue arrow); (**d**) high magnification SEM image of region II showing high density of slip lines on L1_2_ phase at the end of scratch.

To evaluate the difference in friction and pile-up behavior between the two phases, localized scratch test was performed across the alternating lamellae of L1_2_ and B2 phases. The coefficient of friction (COF) of each phase was evaluated by monitoring the material removal mechanism in the SEM. The B2 phase exhibited higher COF as compared with the relatively more ductile L1_2_ phase (Fig. 8(a)). The average COF of B2 was ~0.89 while that of L1_2_ was ~0.83. The inset image of Fig. 8(a) shows the sample and the indenter after completion of the scratch with almost no material adhering to the cube corner diamond probe. The indenter movement was slower in the hard B2 phase with sudden increase in COF compared to the softer L1_2_ phase. An *in situ* scratch video is included as supplementary document. Figure 8(b) shows low magnification SEM image of the scratch with the displaced material ejected out of the wear track at the end. Along the wear track, there was a difference in material pile up behavior between the L1_2_ and B2 phases. There was more pile up or material removal for L1_2_ phase due to its ductile nature and lower hardness, while significantly lower material removal was noticed for B2 phase because of its brittle nature and higher hardness. These two behaviors are indicated with a red arrow for L1_2_ phase and a blue arrow for B2 phase in Fig. 8(c). The L1_2_ phase showed multiple slip lines perpendicular to the scratch direction while there was almost no deformation for the B2 phase as seen in Fig. 8(c). The high density of slip lines for L1_2_ were arrested at the L1_2_/B2 interface as clearly seen in the high magnification image in Fig. 8(d). Deformation under contact behaves elastically for high *H*/E* (hardness/modulus) materials because they are under reduced contact pressure^47^. In the current study, the *H*/E* value was ~0.027 for the B2 phase and ~0.018 for L1_2_ phase. Therefore, the L1_2_ deformed plastically to a larger extent by forming slip lines while the B2 phase showed limited deformation.

## Conclusions

In this study, the mechanical behavior of AlCoCrFeNi_2.1_ eutectic high entropy alloy at small length scales was investigated for fundamental understanding of phase-specific contribution to the bulk mechanical response. The main conclusions are as follows:

1. The as-cast microstructure consisted of eutectic lamellae of: (a) L1_2_ phase rich in Fe, Co, Cr and (b) B2 phase rich in Ni and Al. The average grain size of the cast alloy was ~100 μm. The B2 phase consisted of Cr-rich precipitates with disordered BCC structure and average size of ~20 nm.
2. The AlCoCrFeNi_2.1_ eutectic high entropy alloy showed excellent combination of strength and ductility in tensile and compression loading. In tensile loading, it showed a yield strength of 750 ± 24 MPa, ultimate tensile strength of 1165 ± 20 MPa and elongation of ~18%. In compressive loading, the yield strength was 517 ± 7 MPa, ultimate compressive strength was 1863 ± 12 MPa, and plasticity of ~34.3%. The fracture morphologies confirmed brittle mode for B2 phase and ductile mode for L1_2_ phase.
3. The B2 phase showed higher hardness and slightly lower modulus than L1_2_ phase. Phase specific strain rate sensitivity (SRS) measurements demonstrated higher SRS value for B2 phase compared to L1_2_. The overall SRS value for the alloy was close to that of the B2 phase, indicating B2 controlled deformation of the current EHEA.
4. Uniaxial micro-pillar compression studies showed higher yield strength for B2 phase (~610 MPa) compared to L1_2_ phase (~460 MPa). Mixed pillars containing both the phases showed double yielding phenomenon. TEM analysis at the interface between the two phases showed high density of dislocations in the L1_2_ phase that were arrested by the B2 phase containing high density of Cr-rich precipitates. However, the phase boundary was well preserved during the compression of all mixed pillars even after high compressive strains.
5. The B2 phase showed higher coefficient of friction (COF) compared to the L1_2_ phase. The L1_2_ phase deformed by forming multiple slip lines and significant material pile up during small-scale scratch using a cube corner diamond indenter.

## Supplementary information

Supplementary Information.
